# Low protein diet with personalized support in advanced chronic kidney disease: association with disease progression, dialysis delay and mortality

**DOI:** 10.1093/ckj/sfaf341

**Published:** 2025-11-07

**Authors:** Gisella Vischini, Sara Caissutti, Daniele Vetrano, Sara Donini, Paolo Mastromauro, Anna Laura Chiocchini, Anna Vella, Giacomo Magnoni, Irene Capelli, Giorgina Barbara Piccoli, Gaetano La Manna

**Affiliations:** Nephrology, Dialysis and Kidney Transplant Unit, IRCCS Azienda Ospedaliero-Universitaria di Bologna, Bologna, Italy; Department of Medical and Surgical Science (DIMEC), Alma Mater Studiorum - University of Bologna, Bologna, Italy; Department of Medical and Surgical Science (DIMEC), Alma Mater Studiorum - University of Bologna, Bologna, Italy; Department of Medical and Surgical Science (DIMEC), Alma Mater Studiorum - University of Bologna, Bologna, Italy; Department of Medical and Surgical Science (DIMEC), Alma Mater Studiorum - University of Bologna, Bologna, Italy; Nephrology, Dialysis and Kidney Transplant Unit, IRCCS Azienda Ospedaliero-Universitaria di Bologna, Bologna, Italy; Department of Medical and Surgical Science (DIMEC), Alma Mater Studiorum - University of Bologna, Bologna, Italy; Nephrology, Dialysis and Kidney Transplant Unit, IRCCS Azienda Ospedaliero-Universitaria di Bologna, Bologna, Italy; Nephrology, Dialysis and Kidney Transplant Unit, IRCCS Azienda Ospedaliero-Universitaria di Bologna, Bologna, Italy; Department of Medical and Surgical Science (DIMEC), Alma Mater Studiorum - University of Bologna, Bologna, Italy; Néphrologie et Dialyse, Centre Hospitalier Le Mans, Le Mans, France; Nephrology, Dialysis and Kidney Transplant Unit, IRCCS Azienda Ospedaliero-Universitaria di Bologna, Bologna, Italy; Department of Medical and Surgical Science (DIMEC), Alma Mater Studiorum - University of Bologna, Bologna, Italy

**Keywords:** chronic kidney disease, dialysis, eGFR, malnutrition, pre-dialysis

## Abstract

**Background:**

Despite therapeutic strategies to manage comorbidities and risk factors associated with chronic kidney disease (CKD), a significant number of patients continue to progress toward kidney failure. The role of low protein diets (LPDs) in delaying the need for kidney replacement therapy (KRT) remains a subject of ongoing discussion. Real-world studies offer insights into the risks, implementation and feasibility of LPDs.

**Methods:**

We retrospectively evaluated the efficacy of a moderate LPD (0.6–0.7 g/kg/day) proposed to all newly referred patients with advanced CKD. Survival and need for dialysis were compared between patients who choose an LPD and those who did not. Follow-up was otherwise identical in both groups.

**Results:**

Between January 2021 and December 2023, 110 of 182 patients chose to follow an LPD, while 72 did not. No baseline difference in age, sex, kidney function and comorbidity were observed between the two groups. The decline in estimated glomerular filtration rate was faster in patients not adhering to a diet compared with those who followed it (−2.65 versus −0.89 ml/min/1.73 m^2^/year), with an 84% reduction in the need for KRT {hazard ratio [HR] 0.16 [95% confidence interval (CI) 0.07–0.39]}, 78% in all-cause mortality [HR 0.22 (95% CI 0.06–0.72)] and 81% in the composite outcome death or dialysis [HR 0.19 (95% CI 0.09–0.38)].

**Conclusion:**

When offered the option of an LPD, >60% of patients agreed to and followed it. Choosing an LPD was associated with improved patient and renal survival. While this retrospective study cannot establish a causal relationship, our data provide reassurance regarding feasibility and suggest potential advantages of offering LPDs to unselected patients with CKD who choose this dietary approach.

## GRAPHICAL ABSTRACT

**GRAPHICAL ABSTRACT:**
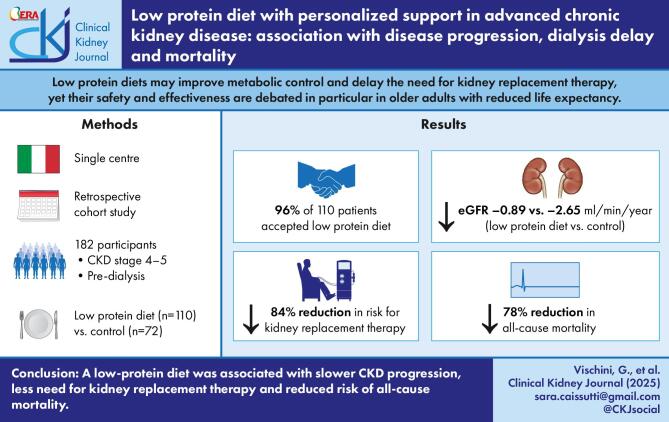

KEY LEARNING POINTS **What was known:**

- The global prevalence of CKD, currently estimated at 8–12%, is increasing due to aging populations and the increasing incidence of comorbidities.
- Delaying dialysis offers a win–win scenario for patients and the healthcare system.
- Concerns about safety and compliance have limited the widespread adoption of nutritional management, despite its potential relevance, particularly in late CKD stages, especially considering that new pharmacological strategies primarily target earlier stages of CKD.

**This study adds:**

- When presented with the choice of a moderately restricted LPD, more than half of the patients opted for it and demonstrated good acceptability.
- In the absence of significant differences in baseline characteristics and follow-up policies, patients who chose to follow an LPD experienced higher survival rates and a lower need for kidney replacement therapy.
- While a causal relationship cannot be inferred from this observational study, this real-world experience suggests that patient empowerment and dietary choice are key factors for positive outcomes in patients with advanced CKD.

**Potential impact:**

- This observational study provides reassurance regarding the safety of moderately restricted LPDs and offers valuable insights into their implementation and impact on hard clinical outcomes in patients who choose this dietary approach.
- Given that no baseline characteristic was associated with the choice of following an LPD, a patient-centred approach emphasizing nutrition education may facilitate the identification of individuals who could benefit from nutritional management in advanced CKD.
- The significantly lower mortality and need for renal replacement therapy found in patients who choose an LPD warrant confirmation in further studies where prescription is based on patient choice.

## INTRODUCTION

Chronic kidney disease (CKD) is estimated to affect ≈850 million people worldwide [1], and this number is projected to increase due to the increasing prevalence of risk factors and the aging of the population. This trend places a significant burden on public health systems, particularly following the initiation of kidney replacement therapy (KRT), and impacts patients’ quality of life. In Europe, >1 million individuals are undergoing KRT, representing >2% of total healthcare expenditures, excluding additional social costs [2–4]. While economic considerations are highly context dependent, healthcare costs increase with disease progression. In Italy, the direct healthcare costs of CKD management range from €1169 in stage 1 to €5453 in stage 5 and increasing to €43 800 for haemodialysis. Estimates vary across Italian regions. Indirect costs associated with CKD are likely underestimated (in Italy €6650/person/year), especially for elderly and frail patients requiring assistance [4]. Delaying dialysis offers benefits not only for the patient but also for the healthcare system [5, 6]. Indeed, patients on dialysis have a significantly shorter life expectancy compared with the general population, with an annual mortality rate of 15–20% and a 5-year survival rate of ≈40% in the elderly populations currently undergoing treatment [7, 8]. The increasing impact of CKD underscores the need for strategies to slow its progression, improve quality of life and prevent disability. Novel therapeutic options target comorbidities, risk factors and disease progression [9]. However, many patients still progress to kidney failure.

A low protein diet (LPD) may aid in managing metabolic complications and delaying the need for KRT. Data regarding the effectiveness of nutritional therapy are conflicting [9–15]. While primarily based on the same series of randomized controlled trials (RCTs), the two main international guidelines addressing nutrition and CKD [Kidney Disease Outcomes Quality Initiative (KDOQI) 2020 and Kidney Disease: Improving Global Outcomes (KDIGO) 2024] [9, 10] provide almost divergent recommendations. The KDOQI strongly recommends (1A) the use of low or very low protein diets (LPDs or vLPDs) from CKD stage 3 onwards, while the KDIGO rates the same evidence level as allowing for a 2C recommendation, acknowledging a role for vLPDs only in highly selected cases and highlighting the risks of low compliance and poor quality of life. Neither guideline adequately considers age, a significant issue given the increasing age of the CKD population, at least in European countries. A joint statement from the European Renal Nutrition (ERN) working group and the European Society for Parenteral and Enteral Nutrition (ESPEN) emphasizes the need for a personalized approach tailored to patients’ clinical status, preferences and needs [16]. Given the conflicting interpretations of the limited RCTs on this topic and the ongoing debate regarding the safety and effectiveness of nutritional approaches, real-world studies are essential.

Unlike RCTs, in clinical practice, patient choice significantly influences outcomes. In this context we retrospectively evaluated the feasibility and main results observed in patients who chose to follow a personalized LPD in our unit in Bologna, Italy, where a moderately protein-restricted diet is considered part of the ‘standard of care’ [17]. The analysis of the results of this patient-centred approach to the choice of LPD may offer valuable insights into implementation strategies and outcomes.

## MATERIALS AND METHODS

### Ethics

As this research involved the retrospective analysis of anonymized/de-identified data collected during routine clinical practice (standard of care) and did not include any intentional interventions beyond standard clinical management, formal ethical committee approval was not mandated. This observational study retrospectively collected data on patients with advanced CKD (stage 4–5 according to the KDIGO) [10] who were followed in our dedicated outpatient unit for at least 6 months between January 2021 and December 2023. All stable patients with no active neoplasia (haematologic or solid) and/or affected by cachexia/malnutrition were offered nutritional therapy involving an LPD (0.6–0.7 g/kg/day), with controlled sodium, phosphorus and energy intake tailored to their individual needs and preferences. The diet, developed by renal nutrition specialists, was personalized based on patient preferences and a 3-day food record and could be designed with or without protein-free products. One ‘unrestricted’ meal per week was permitted to improve acceptability and reduce psychological burden. Acceptability was evaluated as the extent to which self-reported eating behaviour aligned with nutritional recommendations along with estimated protein intake by the Maroni and Mitch formula [18] between the months 3 and 6 of dietary initiation. ‘Personalized support’ was a patient-centred implementation to optimize patient compliance and long-term feasibility. This involved two key mechanisms:

• Continuous feedback loop and assessment of feasibility: regular, one-on-one consultations were conducted to actively seek and incorporate constant feedback from the patient regarding the real-world feasibility of the prescribed diet.
• Personalization of food choices (nutrient equivalency): within the constraints of the prescribed nutrient targets the nutritional team offered personalized food alternative options to align with the patient’s individual taste preferences and cultural background.
 Patients were categorized into two groups: the ‘diet group’ included patients who chose and adhered to an average protein intake of 0.6–0.7 g/kg/day, while the ‘no diet’ group comprised patients who decided not to follow an LPD or were unable to comply with it. Each follow-up visit included blood tests (always encompassing renal function, electrolytes, albumin, lipid profile assessment, haemoglobin, ferritin, iron and bone metabolism, with additional tests as needed) and physical examination to evaluate nutritional status and CKD progression.

All patients were monitored at least every 2–3 months according to their disease trajectory, the severity of kidney function impairment and comorbidity burden. Established targeted questions assessed diet palatability and sustainability, quality of life (subjective well-being, daily activities, autonomy) and acceptability (direct questions, blood urea nitrogen, serum creatinine, albumin levels, lipid assessment and body weight changes) were routinely collected (Supplementary Fig. S2). Dietary therapy was discussed during each visit, emphasizing the importance of adhering to the prescribed diet to stabilize estimated glomerular filtration rate (eGFR) and metabolic control and prevent hypercatabolism and malnutrition. Re-evaluations with dietitians were organized as required.

The study aimed to evaluate the feasibility of the LPD in our setting (measured by the prevalence of patients who chose and correctly followed the diet) and the association between the implementation of nutritional therapy and the progression to kidney failure, defined as the need to initiate dialysis (since no pre-emptive kidney transplantation was recorded in this population), all-cause mortality, non-fatal cardiovascular events and a composite endpoint including all three events. Secondary analyses included the annual slope of eGFR, metabolic complications (uraemia-related symptoms, symptomatic metabolic acidosis) and nutritional status [assessed with serum monitoring of albumin, haemoglobin, total cholesterol levels, body weight and body mass index (BMI)].

### Statistical analysis

Baseline characteristics were summarized as mean ± standard deviation (SD) for normally distributed continuous variables, median and interquartile range (IQR) for non-normally distributed variables and number and percentages for categorical variables. Between-group comparisons were conducted using the unpaired Student’s *t*-test or Wilcoxon rank-sum test for continuous variables, according to distribution, and the chi-squares or Fisher’s exact test for categorical variables.

The median follow-up was estimated using the reverse Kaplan–Meier method. Patients were followed until death, initiation of KRT or the last visit; those lost to follow-up were right-censored.

Event-free survival for the composite outcome (dialysis, death or non-fatal cardiovascular events) and its components was estimated using Kaplan–Meier curves and compared with the logrank test. Cox proportional hazards models (univariate and multivariate) were used to estimate hazard ratios (HRs) and 95% confidence intervals (CIs) for the effect of dietary intervention, adjusting for prespecified covariates: age, sex, eGFR, proteinuria, diabetes, cardiovascular disease, obesity and renin–angiotensin–aldosterone system inhibitor (RAASi) use. Due to the limited number of events, adjustment for covariates was performed progressively across three models. The proportional hazards assumption was verified with Schoenfeld residuals and graphical assessment.

Potential effect modification was evaluated through interaction terms (e.g. group × covariate) in Cox models, with HRs for interaction and corresponding *P*-values. To account for competing risks among outcome components, Fine and Gray’s subdistribution hazard models were used to estimate subdistribution hazard ratios (SHRs) and 95% CIs. Sensitivity analyses excluded patients with extreme baseline values (age <50 or >80 years, eGFR <10 or >20 ml/min/1.73 m^2^, proteinuria <0.3 or >5.0 g/day), with re-estimation of adjusted Cox models.

Longitudinal eGFR changes were analysed using linear mixed-effects models with random intercepts, including fixed effects for time, group and their interaction, and adjustments for age, diabetes, cardiovascular disease and RAASi therapy. eGFR decline was expressed as an annual slope (ml/min/1.73 m^2^/year) with 95% CIs.

Post hoc analyses of bicarbonate, phosphorus, urea and albumin changes at follow-up were conducted and between-group differences at last follow-up were assessed with the unpaired Student’s *t*-test or Wilcoxon rank-sum test.

All analyses were performed in R version 4.4.2 (R Foundation for Statistical Computing, Vienna, Austria), with statistical significance set at *P* < .05.

## RESULTS

**Figure 1:**
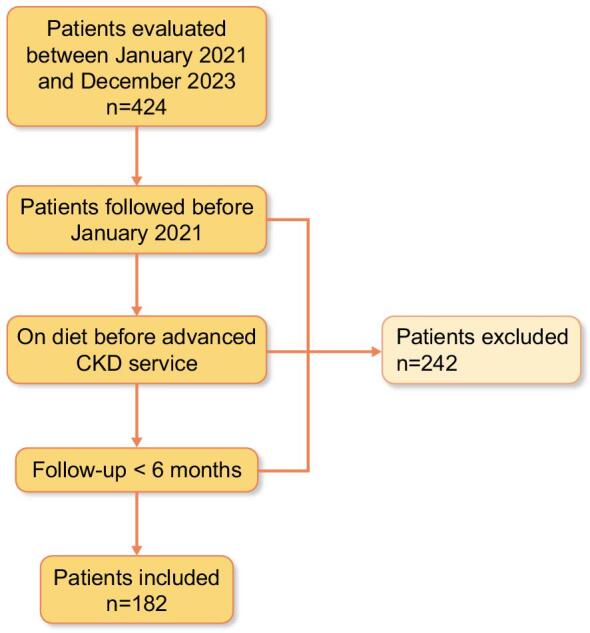
Flow chart of patient selection.

From 424 adult patients followed up in our advanced CKD service from January 2021 to December 2023, 182 patients who initiated follow-up in the unit, were diet-naïve and were followed for at least 6 months were selected (Fig. 1).

Of the 182 selected cases, 110 (60.4%) chose and were able to follow a moderately restricted LPD (diet group), while 72 (39.6%) did not (no-diet group).

Tables 1 and 2 summarizes the baseline characteristics of the study population. The mean age at referral was 71.2 ± 13.6 years, with a similar distribution of sex in both groups. Baseline kidney function parameters, including serum creatinine (3.38 ± 1.05 versus 3.26 ± 0.86 mg/dl; *P* = .41) and eGFR (17.14 ± 5.85 versus 17.75 ± 4.96 ml/min/1.73 m^2^; *P* = .45), as well as proteinuria levels [median urine protein 0.85 g/24 h (IQR 0.36–2.02) versus 0.96 g/24 h (IQR 0.41–2.10); *P* = .17), were similar in both groups. Comorbidities including diabetes, hypertension and cardiovascular diseases were evenly distributed.

**Table 1:** Baseline characteristics of the patients.

Characteristics	All (*N* = 182)	Diet (*N* = 110)	No-diet (*N* = 72)	*P*-value
Age (years), mean (SD)	71.21 (13.58)	69.89 (14.19)	73.22 (12.43)	.106
Male, *n* (%)	119 (65.4)	72 (65.5)	47 (65.3)	.980
Weight (kg), median (IQR)	75.00 (63.00–88.12)	77.00 (65.00–91.00)	71.00 (60.50–80.50)	.054
BMI (kg/m^2^), median (IQR)	27.70 (24.36–32.16)	27.82 (24.62–32.25)	24.45 (22.12–31.65)	.243
Smoker, *n* (%)	55 (30.6)	28 (25.9)	27 (37.5)	.099
Diabetes, *n* (%)	78 (43.3)	48 (44.0)	30 (42.3)	.813
Hypertension, *n* (%)	158 (86.8)	94 (85.5)	64 (88.9)	.503
CVD, *n* (%)	80 (44.0)	49 (44.5)	31 (43.1)	.843
Ischaemic heart disease, *n* (%)	43 (23.6)	25 (22.7)	18 (25.0)	.724
Stroke, *n* (%)	8 (4.4)	4 (3.6)	4 (5.6)	.537
Angina pectoris, *n* (%)	7 (3.8)	5 (4.5)	2 (2.8)	.544
Hearth failure, *n* (%)	23 (12.6)	15 (13.6)	8 (11.1)	.616
Cause of CKD, *n* (%)				.255
Arterionephrosclerosis	34 (18.7)	23 (20.9)	11 (15.3)	
Diabetic nephropathy	11 (6)	9 (8.2)	2 (2.8)	
Glomerulonephritis	7 (3.8)	2 (1.8)	5 (6.9)	
Tubulointerstitial nephritis	12 (6.6)	7 (6.4)	5 (6.9)	
ADPKD	8 (4.4)	6 (5.5)	2 (2.8)	
Unknown	110 (59.9)	63 (56.4)	47 (65.3)	
SCr (mg/dl), mean (SD)	3.31 (0.94)	3.26 (0.86)	3.38 (1.05)	.413
eGFR (ml/min/1.73 m^2^), mean (SD)	17.51 (5.32)	17.75 (4.96)	17.14 (5.85)	.447
Urea (mg/dl), mean (SD)	118.60 (43.21)	119.91 (43.25)	116.60 (43.37)	.614
Sodium (mmol/l), mean (SD)	139.82 (3.01)	139.83 (3.21)	139.81 (2.68)	.978
Potassium (mmol/l), mean (SD)	4.56 (0.55)	4.51 (0.54)	4.64 (0.56)	.121
HbA1c (diabetics) (mmol/mol), mean (SD)	51.90 (14.72)	50.37 (14.44)	55.08 (15.38)	.350
Calcium (mg/dl), mean (SD)	9.18 (0.71)	9.27 (0.72)	9.05 (0.69)	.042
Phosphorus (mg/dl), mean (SD)	4.03 (0.81)	4.02 (0.76)	4.06 (0.87)	.754
PTH (pg/ml), median (IQR)	153.00 (103.00–249.50)	156.00 (112.00–251.75)	149.00 (98.50–238.00)	.671
Uric acid (mg/dl), mean (SD)	6.14 (1.77)	6.04 (1.74)	6.30 (1.83)	.340
Albumin (g/l), mean (SD)	34.73 (12.28)	33.77 (13.85)	36.25 (9.13)	.199
Total cholesterol (mg/dl), median (IQR)	166.00 (132.00–200.00)	167.50 (132.00–198.50)	164.00 (136.50–203.50)	.802
LDL (mg/dl), median (IQR)	92.00 (68.00–121.00)	92.50 (68.65–123.05)	91.00 (61.60–116.00)	.867
TG (mg/dl), median (IQR)	132.00 (99.00–189.00)	137.00 (103.00–190.00)	123.50 (91.75–175.25)	.450
HDL (mg/dl), median (IQR)	45.00 (38.00–53.00)	45.00 (38.00–53.00)	44.00 (38.50–54.50)	.359
Haemoglobin (g/l), mean (SD)	11.77 (1.48)	11.93 (1.47)	11.52 (1.47)	.067
Ferritin (mg/dl), median (IQR)	88.00 (45.00–174.50)	89.50 (47.25–180.00)	80.00 (37.50–153.25)	.925
Diuresis (l/24 h), median (IQR)	1.9 (1.5–2.4)	2.0 (1.5–2.5)	1.80 (1.50–2.3)	.744
Urine protein (g/24 h), median (IQR)	0.94 (0.40–2.10)	0.96 (0.41–2.10)	0.85 (0.36–2.02)	.174
CO_2_ (mmol/l), mean (SD)	22.74 (4.74)	22.05 (3.28)	23.82 (6.26)	.033
HCO_3_ (mmol/l), mean (SD)	23.60 (4.37)	23.52 (3.61)	23.73 (5.37)	.790
SBP (mmHg), mean (SD)	138.82 (20.70)	141.68 (21.36)	134.50 (19.00)	.024
DBP (mmHg), mean (SD)	78.94 (12.18)	80.14 (12.94)	77.14 (10.79)	.111
Heart rate (bpm), mean (SD)	69.62 (10.30)	68.97 (11.38)	70.49 (8.73)	.448

ADPKD: autosomal dominant polycystic kidney disease; HTN: hypertension; CVD: cardiovascular disease; SCr: serum creatinine; HbA1c: haemoglobin A1c; PTH: parathyroid hormone; LDL: low-density lipoprotein; TG: triglycerides; HDL: high-density lipoprotein; CO_2_: carbon dioxide; HCO_3_, bicarbonate; SBP: systolic blood pressure; DBP: diastolic blood pressure.

**Table 2:** Drug therapy at baseline.

Characteristics	All (*N* = 182)	Diet (*N* = 110)	No diet (*N* = 72)	*P*-value
Total drugs, median (IQR)	8.00 (6.00–10.00)	8.00 (6.00–10.00)	8.00 (7.00–10.00)	.662
Antihypertensive drugs, median (IQR)	2.00 (2.00–3.00)	2.00 (2.00–3.00)	2.00 (2.00–3.00)	.854
RAASi, *n* (%)	53 (29.1)	34 (30.9)	19 (26.4)	.512
MRA, *n* (%)	12 (6.6)	7 (6.4)	5 (6.9)	.877
Diuretics, *n* (%)	93 (51.4)	52 (47.7)	41 (56.9)	.224
ESA, *n* (%)	71 (39.0)	38 (34.5)	33 (45.8)	.127
VDRA, n (%)	87 (47.8)	50 (45.5)	37 (51.4)	.433
Phosphorus binders, *n* (%)	17 (9.4)	6 (5.5)	11 (15.3)	.027
Potassium binders, *n* (%)	18 (9.9)	12 (10.9)	6 (8.3)	.569
Oral bicarbonate, *n* (%)	48 (26.4)	30 (27.3)	18 (25.0)	.734
Insulin, *n* (%)	44 (24.2)	24 (21.8)	20 (27.8)	.359
OAD, *n* (%)	41 (22.5)	28 (25.5)	13 (18.1)	.243

MRA: mineralocorticoid receptor antagonist; ESA: erythropoiesis-stimulating agent; VDRA: Vitamin D receptor agonist; OAD: oral antidiabetic drug.

The median follow-up was 18 months (IQR 15–28). No patient received pre-emptive kidney transplant. In the on-diet group, the prescribed protein intake was maintained at a high rate of 95.4% throughout the study. Using the Maroni and Mitch formula, the median daily protein intake was 0.72 ± 0.15 g/day for 58 of the 110 patients. In contrast, the median intake for 23 of the 72 patients in the control group was 0.88 ± 0.24 g/day (*P* < .001).

**Figure 2:**
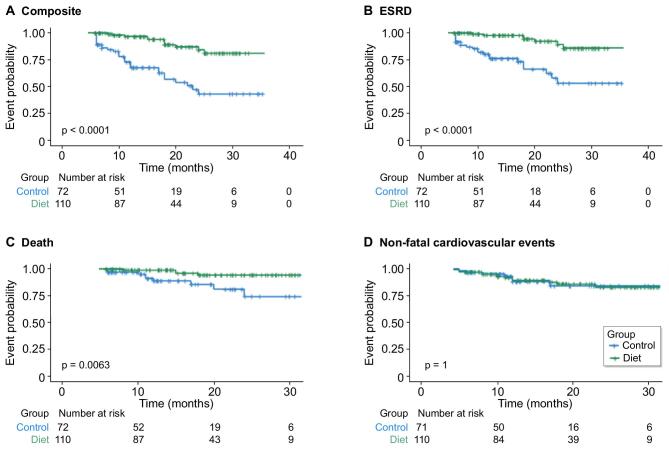
Kaplan–Meier curves of outcome probability stratified by treatment group: **(A)** probability of the composite event (ESRD, all-cause mortality, non-fatal cardiovascular events); **(B)** probability of reaching ESRD, defined by the need to start dialysis treatment; **(C)** all-cause mortality; **(D)** probability of non-fatal cardiovascular events.

The on-diet group showed a significantly lower risk of reaching the composite endpoint (*P* < .0001), with a 12-month event-free survival of 85.5% versus 62.3% in the no-diet group [crude HR 0.19 (95% CI 0.09–0.38)] (Fig. 2A).

Kaplan–Meier curves indicated a lower incidence of dialysis need [*P* < .0001; 12-month event-free survival 90.3% versus 71.2%, crude HR 0.16 (95% CI 0.07–0.39)] and all-cause mortality [*P* = .0063; 12-month survival 93.2% versus 82.4%, crude HR 0.22 (95% CI 0.06–0.72)] in the diet group. No significant difference was found in non-fatal cardiovascular events (*P* = 1.00) (Fig. 2D).

After adjusting for age, sex, smoking status, baseline diabetes, cardiovascular disease, BMI >30 kg/m^2^, eGFR, proteinuria and RAASi therapy, the choice of an LPD remained associated with a lower incidence of the composite outcome, dialysis initiation and all-cause mortality, but not with cardiovascular outcomes (Table 3).

**Table 3:** Cox models assessing the effect of the diet group on outcomes with incremental adjustments: model 1 adjusted for age, sex and smoking status; model 2 adjusted for baseline diabetes, CV disease and BMI >30; and model 3 adjusted for baseline eGFR, proteinuria and RAASi therapy.

Effect	Crude		Model 1		Model 2		Model 3	
Outcome	HR	95% CI	HR	95% CI	HR	95% CI	HR	95% CI
Composite	0.19	0.09–0.38	0.19	0.09–0.39	0.19	0.07–0.54	0.13	0.05–0.32
ESRD	0.16	0.07–0.39	0.16	0.06–0.39	0.18	0.05–0.65	0.12	0.04–0.34
All-cause mortality	0.22	0.06–0.72	0.26	0.07–0.86	0.23	0.04–0.93	0.15	0.03–0.77
Non-fatal CV events	1.00	0.39–2.52	1.08	0.42–2.77	0.96	0.20–4.46	1.09	0.36–3.27

CV: cardiovascular.

During the follow-up of 182 patients, 13 (7.1%) fatalities were observed. Specifically, four deaths occurred in the on-diet group (median age 72 ± 4 years) and nine in the control group (median age 81 ± 9 years). Cardiovascular events represented the main cause of death in seven of these patients (two patients out of four in on-diet group), while neoplastic diseases accounted for the others (four patients out of nine in the control group, no death for neoplastic disease in the on-diet group).

**Figure 3:**
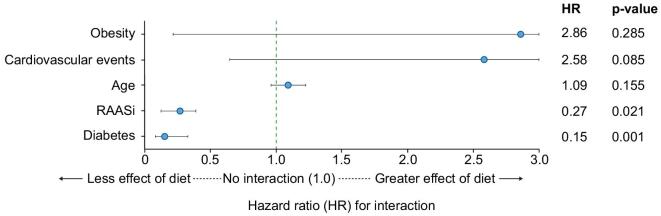
Forest plot of interaction HRs for the primary composite outcome. An HR for interaction <1 suggests a weaker effect of the diet in that subgroup, whereas an HR >1 suggests a stronger effect. HR and *P*-value are reported on the right side.

Interaction analyses, examining the relationship of baseline characteristics including age, obesity, diabetes, cardiovascular diseases and RAASi therapy on the primary composite outcome (Fig. 3 and Supplementary Table S1) revealed no significant interaction between the choice of the diet and age (*P* = .155), obesity (*P* = .285) or cardiovascular events (*P* = .085). Interestingly, a significant interaction was found between following the diet and being affected by diabetes (*P* = .001) or receiving RAASi therapy (*P* = .021), suggesting being on an LPD may be associated with better outcomes in patients without diabetes or without RAASi therapy.

**Figure 4:**
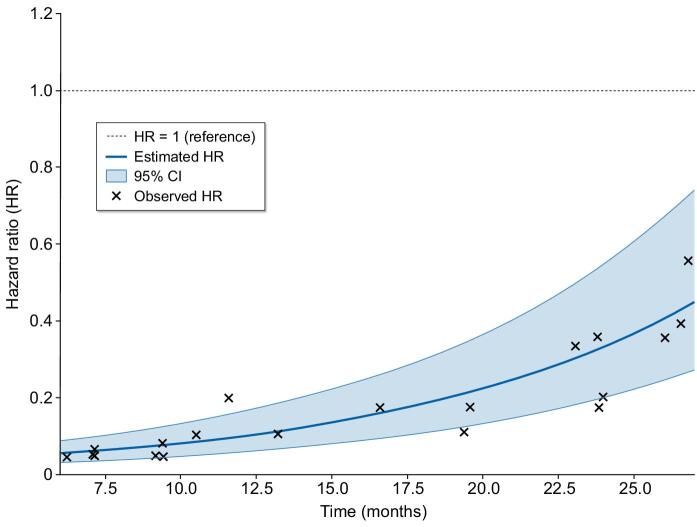
Time-varying Cox proportional HR for the association of the diet group on outcomes.

Figure 4 depicts the Schoenfeld residuals for the primary composite outcome, stratified by the diet group. The graphical representation shows that the risk reduction is consistent over time, with a sustained risk reduction in the diet group.

In the competing risk analysis, the diet group showed a significantly lower subdistribution hazard for end-stage renal disease (ESRD) [SHR 0.158 (95% CI 0.071–0.352), *P* < .001], while no significant differences were observed for non-fatal cardiovascular events [SHR 0.921 (95% CI 0.296–2.87), *P* = .89] or all-cause mortality [SHR 0.323 (95% CI 0.062–1.69), *P* = .18). These findings reinforce the protective effect of the dietary intervention against progression to ESRD but suggest that the mortality benefit observed in the Cox models may be partially mediated by delayed kidney disease progression (Supplementary Table S2).

Sensitivity analyses were conducted to evaluate the robustness of our findings under different inclusion criteria (Supplementary Table S3). Specifically, we excluded patients at the extreme age ranges (<50 or >80 years), those with baseline eGFR values outside 10–20 ml/min/1.73 m^2^ and those with proteinuria levels outside 0.3–5.0 g/day. The results remained consistent across all analyses.

**Figure 5:**
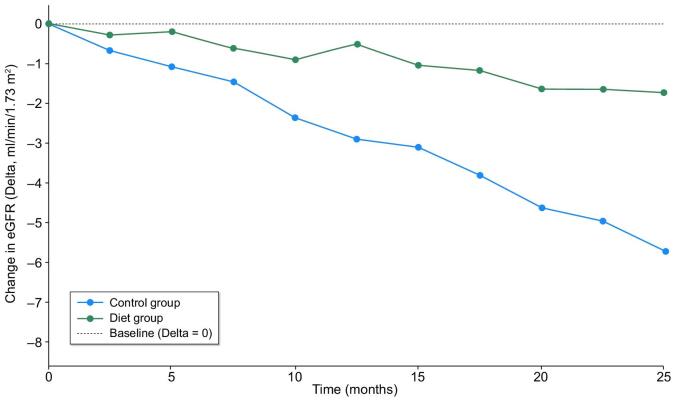
Estimated annual decrease in eGFR (ml/min/1.73 m^2^/year) in the control and diet groups as assessed by linear mixed-effects models.

The difference in GFR decline between patients who followed a diet and those who did not was analysed using linear mixed models (Fig. 5), accounting for repeated measures over time and adjustment for baseline covariates (age, diabetes, cardiovascular disease and RAASi therapy).

The no-diet group exhibited a faster rate of GFR decline, with a median annual slope of −2.65 ml/min/1.73 m^2^/year (95% CI −3.18 to −2.11). In contrast, the diet group experienced a slower rate of decline, with a median annual slope of −0.89 ml/min/1.73 m^2^/year (95% CI −1.52 to −0.26). The difference in slopes between the two groups was −1.77 ml/min/1.73 m^2^/year (95% CI −2.73 to −0.80; *P* = .0004) (Table 4).

**Table 4:** Estimated annual decrease in eGFR (ml/min/1.73 m^2^/year) in the control and diet groups, as assessed by linear mixed-effects models.

Parameter	Unadjusted model	Adjusted model
No-diet group eGFR decline rate (ml/min/1.73 m^2^)	−2.65 (95% CI −3.18 to −2.11)	−2.67 (95% CI −3.20 to −2.14)
Diet group eGFR decline rate (ml/min/1.73 m^2^)	−0.89 (95% CI −1.52 to −0.26)	−0.88 (95% CI −1.51 to −0.25)
Mean difference	−1.77 (95% CI −2.73 to −0.80)	−1.79 (95% CI −2.75 to −0.83)
*P*-value	.0004	.004

Results are shown for both the unadjusted and adjusted models. The adjusted model includes baseline covariates (age, diabetes, cardiovascular disease and RAASi therapy) as fixed effects. The diet group showed a significantly slower decline in kidney function in both models.

Tables 5 and 6 highlights the differences in biochemical parameters and drug therapy between the diet and control groups during follow-up. At the last follow-up, the diet group exhibited a higher serum bicarbonate (24.8 ± 3.7 versus 21.4 ± 4.1 mmol/L; *P* = .014) and haemoglobin levels (12.1 ± 1.3 versus 11.6 ± 1.4 g/dl; *P* = .038), reduced need for erythropoiesis-stimulating agents (36% versus 52%; *P* = .029), lower blood urea (85 ± 22 versus 94 ± 25 mg/dl; *P* = .017) and serum phosphorus levels (3.8 ± 0.6 versus 4.0 ± 0.7 mg/dl; *P* = 0.049), with higher calcium levels (9.2 ± 0.4 versus 8.9 ± 0.5 mg/dl; *P* = .031). Patients who followed an LPD reported a good perception of their quality of life and maintained daily activities, denying fatigue or weakness and reporting good energy levels.

**Table 5:** Anthropomorphic and biochemical parameters between the diet and no-diet groups at last follow-up visit.

Characteristics	All (*N* = 182)	Diet (*N* = 110)	No diet (*N* = 72)	*P*-value
Weight (kg), median (IQR)	74.45 (61.00–87.88)	76.60 (65.00–90.00)	69.00 (59.00–80.00)	.025
BMI (kg/m^2^), median (IQR)	27.15 (23.62–31.34)	27.99 (23.87–32.00)	24.16 (21.45–31.00)	.180
SCr (mg/dl), mean (SD)	4.03 (1.72)	3.76 (1.31)	4.45 (2.14)	.007
eGFR (ml/min/1.73 m^2^), mean (SD)	15.16 (6.55)	15.91 (6.34)	14.01 (6.74)	.016
Urea (mg/dl), mean (SD)	127.37 (44.54)	121.55 (37.39)	136.26 (52.72)	.029
Sodium (mmol/l), mean (SD)	138.93 (14.13)	139.11 (13.38)	138.66 (15.31)	.837
Potassium (mmol/l), mean (SD)	4.69 (2.84)	4.45 (0.48)	5.06 (4.48)	.158
Fasting glucose (mg/dl), median (IQR)	90.50 (80.00–108.00)	90.50 (77.00–111.00)	90.50 (83.00–107.25)	.849
HbA1c (diabetics) (mmol/mol), mean (SD)	47.61 (16.10)	46.79 (11.13)	49.00 (22.70)	.379
Calcium (mg/dl), mean (SD)	9.23 (0.65)	9.35 (0.61)	9.04 (0.66)	.001
Potassium (mg/dl), mean (SD)	4.19 (1.07)	4.04 (0.78)	4.45 (1.39)	.014
PTH (pg/ml), median (IQR)	154.00 (110.00–252.25)	153.50 (116.00–247.75)	157.00 (93.75–255.00)	.359
Uric acid (mg/dl), mean (SD)	6.44 (14.13)	5.17 (1.34)	8.38 (22.36)	.140
Albumin (g/l), mean (SD)	34.87 (11.96)	33.83 (13.43)	36.51 (9.05)	.149
Total cholesterol (mg/dl), median (IQR)	156.50 (128.00–186.00)	158.50 (130.75–184.25)	146.00 (123.25–186.25)	.634
LDL (mg/dl), median (IQR)	79.90 (59.95–106.10)	85.60 (62.85–111.00)	68.50 (55.55–89.75)	.002
TG (mg/dl), median (IQR)	120.50 (96.50–159.00)	121.00 (97.75–160.50)	118.50 (92.75–153.00)	.368
HDL (mg/dl), median (IQR)	48.00 (39.50–55.00)	48.00 (40.75–56.25)	47.00 (38.00–53.00)	.824
Haemoglobin (g/l), mean (SD)	11.81 (1.51)	12.05 (1.55)	11.43 (1.37)	.006
Ferritin (mg/dl), median (IQR)	90.00 (53.00–161.00)	83.00 (52.00–159.50)	93.00 (53.00–167.00)	.808
Diuresis (l/24 h), median (IQR)	1.8 (1.5–2.2)	1.9 (1.6–2.1)	1.80 (1.5–2.4)	.912
Urine protein (g/24 h), median (IQR)	0.90 (0.40–2.70)	0.93 (0.39–2.57)	0.90 (0.40–2.80)	.453
CO_2_ (mmol/l), mean (SD)	22.92 (3.17)	23.28 (2.90)	22.34 (3.50)	.111
HCO_3_ (mmol/l), mean (SD)	24.25 (3.28)	24.78 (2.85)	21.49 (3.72)	.027
SBP (mmHg), mean (SD)	137.76 (20.40)	138.44 (19.37)	136.74 (21.97)	.584
DBP (mmHg), mean (SD)	77.27 (10.55)	77.61 (10.33)	76.74 (10.91)	.585
Heart rate (bpm), mean (SD)	70.28 (9.28)	68.79 (9.31)	72.40 (8.91)	.030

SCr: serum creatinine; HbA1c: haemoglobin A1c; PTH: parathyroid hormone; LDL: low-density lipoprotein; TG:, triglycerides; HDL: high-density lipoprotein; CO_2_: carbon dioxide; HCO_3_: bicarbonate; SBP: systolic blood pressure; DBP: diastolic blood pressure.

**Table 6:** Drug therapy between the diet and no-diet groups at the last follow-up visit.

Therapy	All (*N *= 182)	Diet (*N* = 110)	No diet (*N* = 72)	*P*-value
Total drugs, median (IQR)	10.00 (8.00–12.00)	10.00 (8.00–12.00)	11.00 (8.00–12.50)	.892
RAASi, *n* (%)	36 (19.8)	26 (23.6)	10 (13.9)	.106
RAASi discontinued, *n* (%)	23 (43.4)	13 (38.2)	10 (52.6)	.311
MRA, *n* (%)	7 (3.8)	4 (3.6)	3 (4.2)	.856
Diuretics, *n* (%)	115 (63.2)	68 (61.8)	47 (65.3)	.636
ESA, *n* (%)	94 (52.5)	47 (43.5)	47 (66.2)	.003
VDRA, *n* (%)	96 (53.0)	61 (56.0)	35 (48.6)	.332
Phosphorus binders, *n* (%)	56 (30.9)	30 (27.5)	26 (36.1)	.221
Potassium binders, *n* (%)	45 (24.9)	29 (26.6)	16 (22.2)	.504
Oral bicarbonate, *n* (%)	84 (46.4)	50 (45.9)	34 (47.2)	.858
Insulin, *n* (%)	46 (25.4)	26 (23.9)	20 (27.8)	.553
OAD, *n* (%)	37 (20.4)	25 (22.9)	12 (16.7)	.306

MRA: mineralocorticoid receptor antagonist; ESA: erythropoiesis-stimulating agent; VDRA: vitamin D receptor activator; OAD: oral antidiabetic drug.

## DISCUSSION

The focus on patient-centred approaches highlights patients’ choice as the foundation for establishing a therapeutic strategy. In line with this, in our unit dedicated to the follow-up of patients with advanced CKD, which predominantly manages relatively older patients (mean age 72 years) with significant comorbidity (>40% with diabetes or cardiovascular comorbidity; Table 1), we routinely discuss the option of an LPD as a means to delay dialysis initiation. We prescribe it, using a personalized approach and with the support of a trained nutritionist, to patients who wish to adopt it. Apart from the dietary prescriptions, the remainder of the follow-up (drug therapy, frequency of visits) adheres to standard protocols.

More than 60% of the patients (110/182) chose to undergo a moderately reduced LPD and were able to follow it, a finding in line with previous Italian experiences, a country in which nutritional management is a part of the standard of care for patients affected by advanced CKD [19, 20].

The main result of the present observation real-world study, analysing a cohort of 182 diet-naïve patients with advanced CKD (stage 4–5 according to the KDIGO), is that the individuals who chose and followed an LPD experienced a lower risk of starting KRT and a lower mortality rate. Indeed, we observed an 84% reduction in the incidence of KRT over the study period, without an attrition bias with mortality (which, in contrast, shows a 78% reduction). No differences were found in terms of non-fatal cardiovascular events, highlighting the challenge of counteracting, within a few months, the prolonged effect of cardiovascular risk factors accumulating over time (Fig. 3, Table 4). Moreover, in competing risk analysis, the diet group exhibited a significantly reduced subdistribution hazard for ESRD, contrasting with no significant effects on non-fatal cardiovascular events or all-cause mortality. This reinforces the diet’s protective role against ESRD progression, implying the mortality benefit observed in Cox models may be partially driven by delayed kidney disease progression.

Due to the observational nature of the study, it is impossible to attribute these impressive differences to the LPD itself, or to the fact that patients who chose dietary treatment are expected to have overall better compliance that probably encompasses all treatment aspects and reflects greater attention to a healthy lifestyle.

Together with stable albumin, haemoglobin and total cholesterol levels, body weight, BMI and drug burden, our data provide reassurance regarding the risk of malnutrition, consistent with other studies [19, 21–23] (Tables 5 and 6).

In our study, the annual decrease in eGFR was 2.65 ml/min/1.73 m^2^ in the controls, similar to several experiences in the literature, and 0.89 ml/min/1.73 m^2^ in the diet group, with a significant difference between the two groups (Table 4). Our overall data are in keeping with the median decline of 3.2 ml/min/1.73 m^2^ reported in the last decade [24]. As expected, patients in the diet group showed better metabolic control, particularly of metabolic acidosis, and lower urea levels.

Furthermore, in keeping with the good acceptability, patients who followed an LPD had lower phosphorus levels without differences in parathyroid hormone and calcium levels and lipid and iron profiles. Of note, haemoglobin levels were significantly higher in the on-diet group, with reduced need for erythropoietin therapy, presumably linked to the anti-inflammatory effects of diets that include more vegetable servings [25].

While we offered the same nutritional approach to diabetic and non-diabetic patients, the interaction analysis showed that the effects on the composite outcome were lower in diabetic patients; however, poor glycaemic control or significant metabolic discrepancies were not observed in our cohort. The effect on the composite outcome was also less pronounced in patients on RAASi therapy, possibly because this pharmacological category targets the same pathological pathways as the LPD. However, these observations need further confirmation in a larger cohort.

A last relevant point is the lack of baseline clinical differences between patients who do or do not accept following dietary prescriptions. This finding, in keeping with a previous Italian study [26], underlines that in the absence of elements allowing for an *a priori* selection of the cases that will successfully follow a diet, dietary management should be proposed to all CKD patients.

Our study has several limitations that should be acknowledged, including the retrospective design and the lack of propensity score matching between the diet and control groups. However, the baseline clinical characteristics of both groups were well balanced, with no significant differences in kidney function (eGFR, proteinuria), metabolic markers and prevalence of major comorbidities like diabetes and cardiovascular diseases. Interaction analyses showed consistent results across subgroups, supporting the robustness of the associations. Sensitivity analyses, excluding patients with extreme values of age, eGFR and proteinuria, confirmed the persistence of associations between having chosen an LPD and the outcomes across various clinical scenarios. Finally, linear mixed models allowed the evaluation of longitudinal changes in renal function, corroborating the slower eGFR decline in the diet group. Another limitation is the assessment of data on drug and diet treatment adherence, which was self-reported, possibly introducing recall bias. However, the use of a diet diary is validated and is felt by several authors to be more practical in elderly populations, in which 24-h urine collections may be critical [11]. The outcomes were measured over a relatively short follow-up period, which may not fully capture the long-term effects of the dietary intervention and specific tests to monitor protein intake were not available for all patents and were not routinely tested in those who refuse the dietary regimen. Lastly, the study was conducted in a single centre, suggesting that the external validity of the findings should be tested in broader populations with advanced CKD, a limit that is partly counterbalanced by the homogeneity of the follow-up offered to all patients, regardless of their choice.

Despite these limitations, our study presents significant strengths. First, it reflects real-life nephrology practice and addresses common challenges in managing patients with advanced CKD. Second, our work suggests that an LPD is associated with slower CKD progression, delayed need for dialysis and lower mortality. The global approach encompassing LPDs was safe and effective in this high-risk, elderly cohort and we hold that this is due to personalized, intensive follow-up. We strongly believe that patient empowerment, leading them to freely choose whether or not to integrate an LPD into their treatment plan was a key for success.

Further prospective studies with precise assessments of dietary adherence are needed to confirm these results and further understand the pros and cons of nutritional therapy in managing patients with advanced CKD.

## CONCLUSION

The present real-world experience shows that the implementation of an LPD is feasible in >60% of patients with advanced CKD and is associated with better hard outcomes over the follow-up, especially progression to ESRD.

While an observational study, following patients allowed to choose whether or not to be prescribed a protein-restricted diet, cannot conclude on efficacy, it can provide reassurance regarding safety. Furthermore, the lack of baseline differences between individuals who do and do not follow a diet suggests that this option should be extensively discussed with all patients.

## Supplementary Material

sfaf341_Supplemental_Files

## Data Availability

The data underlying this article are available in the article.
