# Supplementary material for: Low protein diet with personalized support in advanced chronic kidney disease: association with disease progression, dialysis delay and mortality
Source: Clin Kidney J. 2025 Nov 7;19(1):sfaf341. doi: 10.1093/ckj/sfaf341 (PMC12836100; doi:10.1093/ckj/sfaf341)

**Supplementary Materials**

**Supplementary table 1 – Interaction Effects of Variables with the Diet Group on the Primary Outcome.**

| **Interaction Variable** | **HR (95% CI)** | **p-value** |
| --- | --- | --- |
| Age | 1.09 (0.96 - 1.23) | 0.155 |
| Obesity | 2.86 (0.22 - 7.42) | 0.285 |
| Diabetes | 0.15 (0.08 - 0.33) | 0.001 |
| Cardiovascular Events | 2.58 (0.64 – 6.25) | 0.085 |
| RAASi | 0.27 (0.12 - 0.39) | 0.021 |

**Supplemetary Table 2. Sub-distribution Hazard Ratio (SHR) and 95% Confidence Intervals (CI) for each component of the composite outcome (ESKD, death, non-fatal cardiovascular event), analyzed using Fine-Gray regression. The diet group shows a significant reduction in the cumulative risk of ESKD, while no significant differences are observed for the other components.**

| **Components** | **SHR** | **95% CI** | **p-value** |
| --- | --- | --- | --- |
| ESKD | 0.158 | 0.071 – 0.352 | <0.001 |
| Death | 0.323 | 0.062 – 1.69 | 0.18 |
| Non-fatal CV event | 0.921 | 0.296 – 2.87 | 0.89 |

**Supplementary table ~~2~~ 3– Sensitivity Analyses for the Effect of the Diet Group on Outcomes**

|  | **Crude** | | **Sensitivity 1** | | **Sensitivity 2** | | | **Sensitivity 3** | |
| --- | --- | --- | --- | --- | --- | --- | --- | --- | --- |
| **Outcome** | **HR** | **95% CI** | **HR** | **95% CI** | | **HR** | **95% CI** | **HR** | **95% CI** |
| Composite | 0.19 | 0.09-0.38 | 0.17 | 0.07-0.40 | | 0.19 | 0.09-0.43 | 0.15 | 0.05-0.44 |
| ESRD | 0.16 | 0.07-0.39 | 0.13 | 0.05-0.37 | | 0.16 | 0.06-0.42 | 0.13 | 0.03-0.47 |
| All-cause mortality | 0.22 | 0.06-0.72 | 0.29 | 0.07-1.25 | | 0.26 | 0.37-1.13 | 0.18 | 0.03-0.97 |
| Non-fatal CV events | 1.00 | 0.39-2.52 | 2.20 | 0.32-3.29 | | 0.75 | 0.16-3.48 | 1.03 | 0.46-2.57 |

Sensitivity 1: Excluding patients aged over 80 years and under 50 years; Sensitivity 2: Excluding patients with eGFR higher than 20 ml/min/1.73m² and lower than 10 ml/min/1.73m²; Sensitivity 3: Excluding patients with proteinuria higher than 5.0 g/day and lower than 0.3 g/day.

**Supplementary Figure 1 -** **Bar Plots with Standard Errors Showing Longitudinal Changes in Key Biochemical Parameters Between Diet and Control Groups**

***
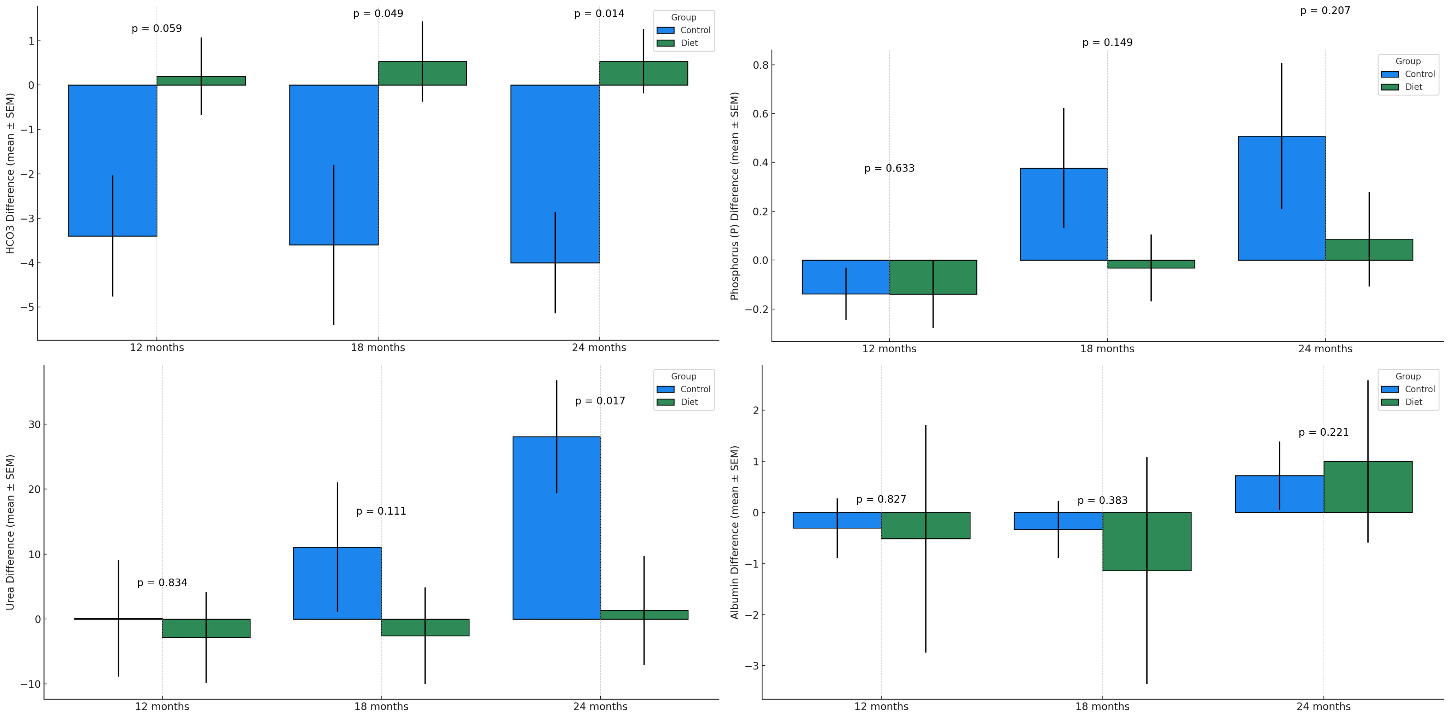
***

**Supplementary Fig.2. Target questions for patients assessment during each visit**


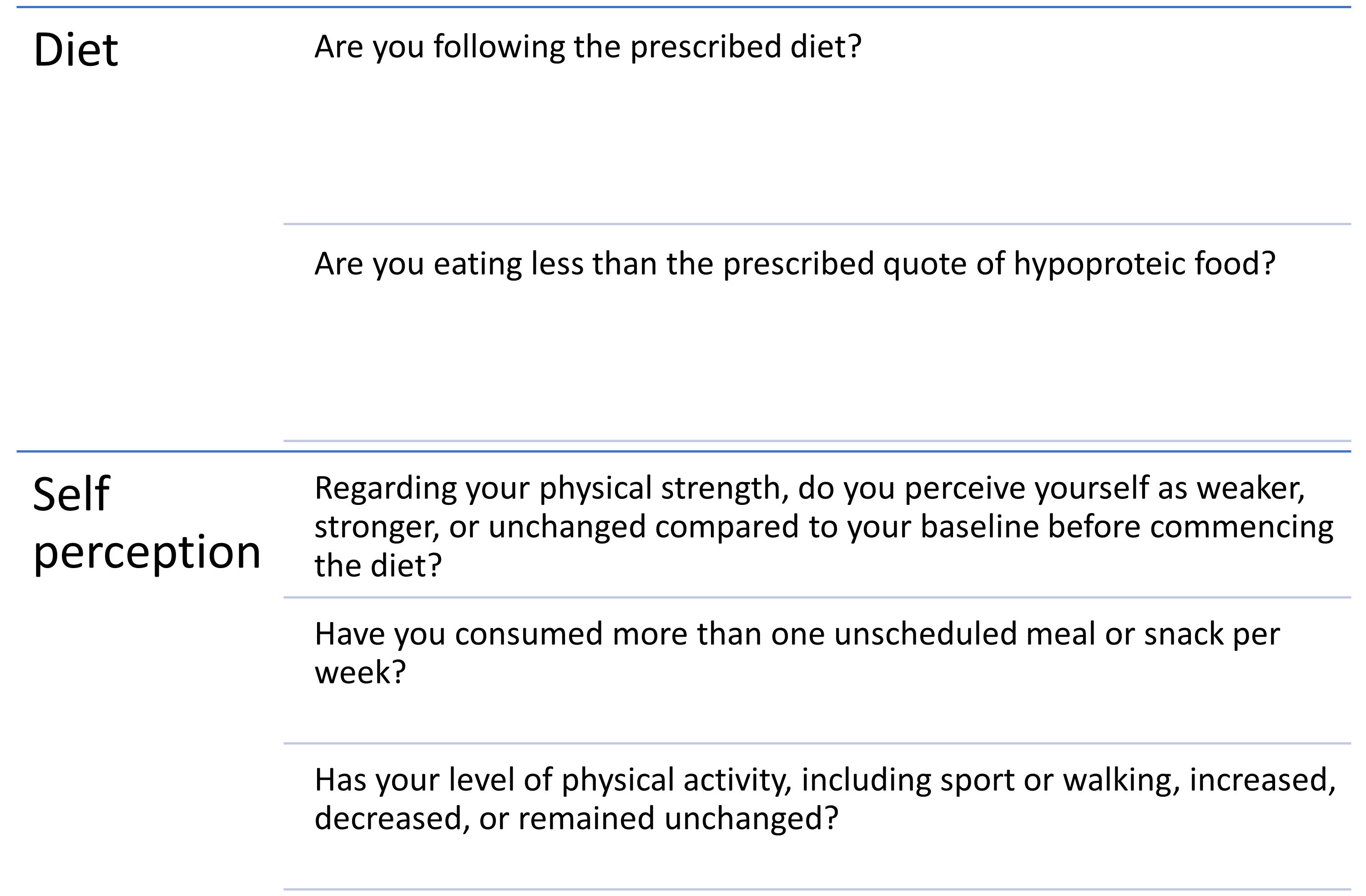

Supplement: sfaf341_Supplemental_Files [file sfaf341_Supplemental_Files.zip › supplementary materials.docx]
