# Supplementary figures and images for: Low protein diet with personalized support in advanced chronic kidney disease: association with disease progression, dialysis delay and mortality
Source: Clin Kidney J. 2025 Nov 7;19(1):sfaf341. doi: 10.1093/ckj/sfaf341 (PMC12836100; doi:10.1093/ckj/sfaf341)

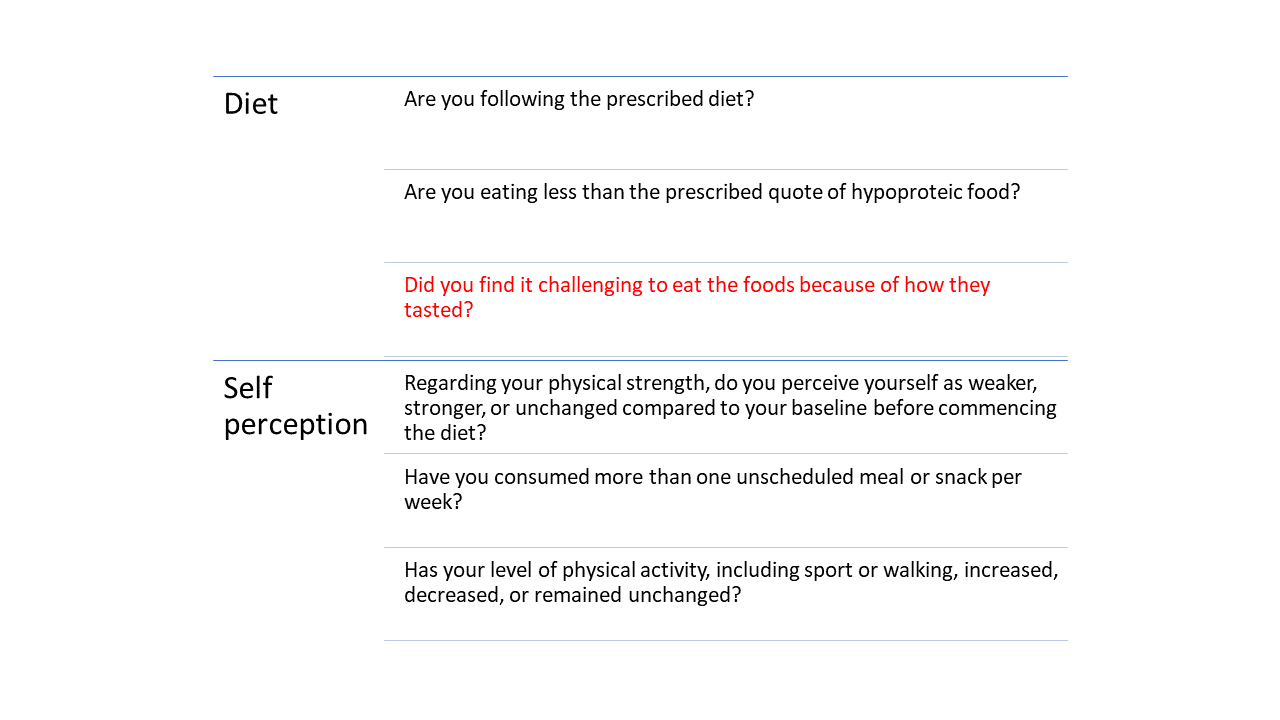

Supplement: sfaf341_Supplemental_Files [file sfaf341_Supplemental_Files.zip › supp Fig2 edited.png]
